# Supplementary figures and images for: Alien species revises systematic status: integrative species delimitation of two similar taxa of Symbrenthia Hübner, [1819] (Lepidoptera, Nymphalidae)
Source: PeerJ. 2023 Jan 30;11:e14644. doi: 10.7717/peerj.14644 (PMC9893915; doi:10.7717/peerj.14644)

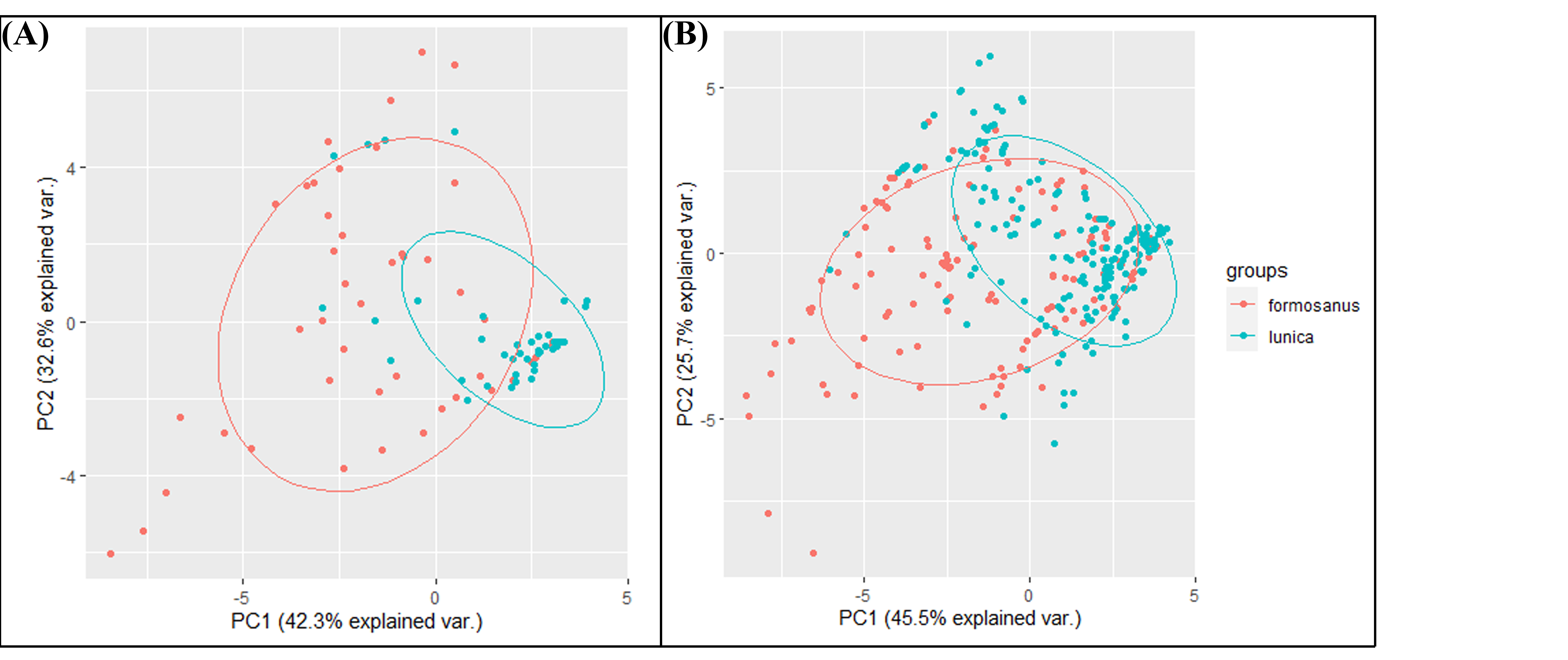

Supplement: Supplemental Information 1 — (A) The niche overlap results of the early invasive stage. (B) The niche overlap results of the late invasive stage. [file peerj-11-14644-s001.png]

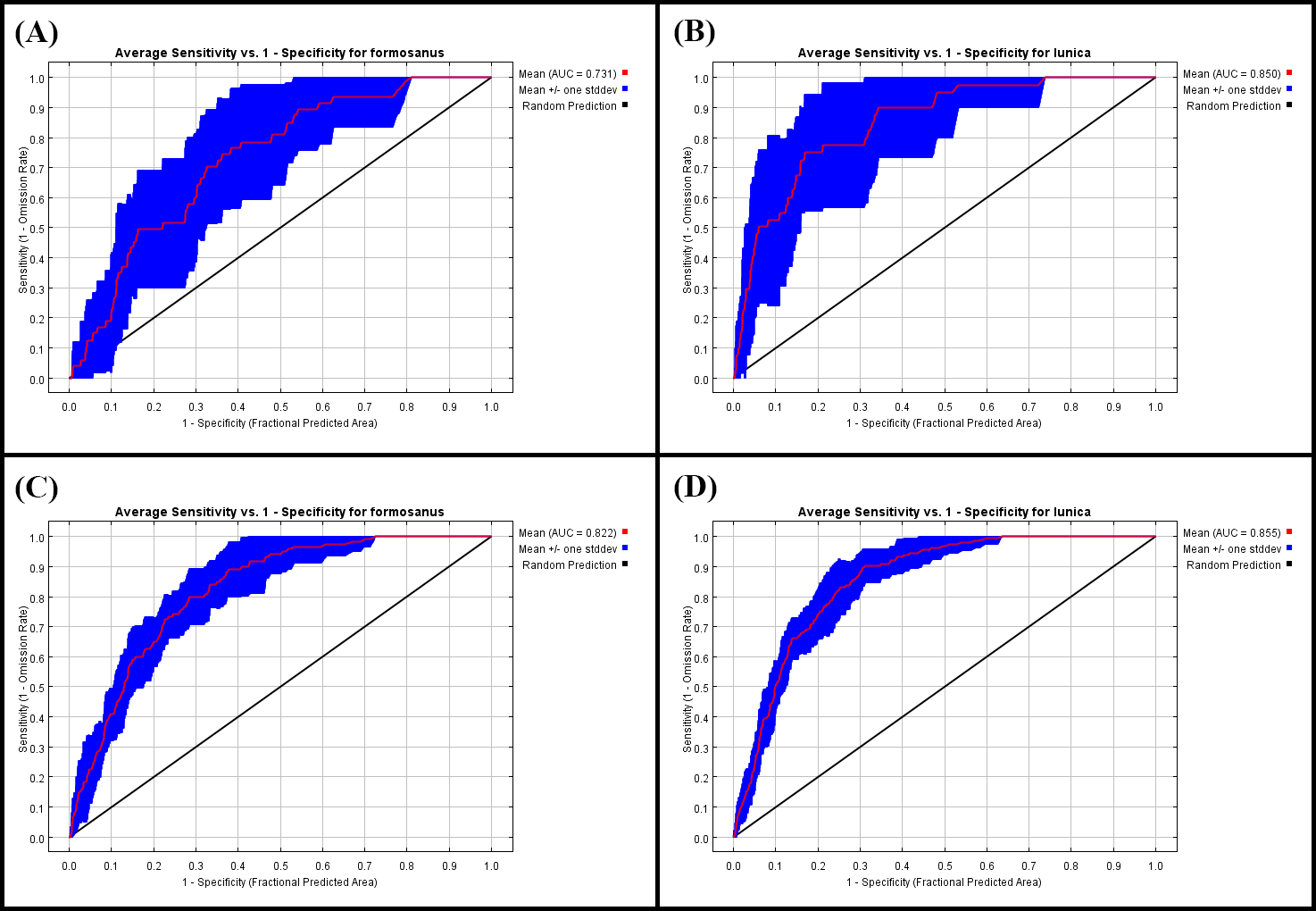

Supplement: Supplemental Information 2 — (A) the ROC and AUC of S. formosanus in the early invasive stage. (B) the ROC and AUC of S. lilaea (=lunica) in the early invasive stage. (C) the ROC and AUC of S. formosanus in the late invasive stage. (D) the ROC and AUC of S. lilaea (=lunica) in the late invasive stage. [file peerj-11-14644-s002.png]

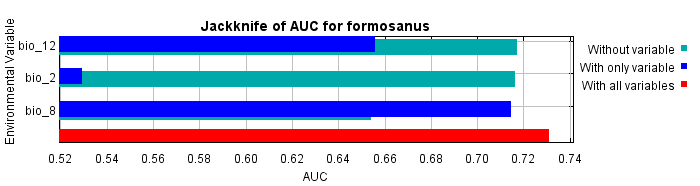

Supplement: Supplemental Information 6 [file peerj-11-14644-s006.zip › SDM/early_stage/MAXENT/formosanus_jacknife_auc.png]

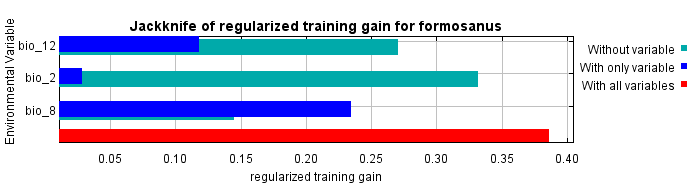

Supplement: Supplemental Information 6 [file peerj-11-14644-s006.zip › SDM/early_stage/MAXENT/formosanus_jacknife_past_2012.png]

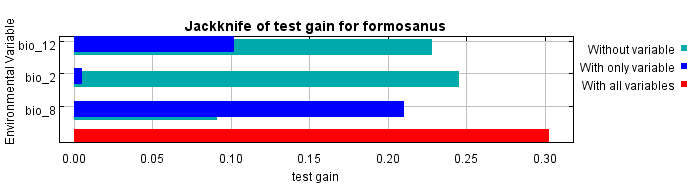

Supplement: Supplemental Information 6 [file peerj-11-14644-s006.zip › SDM/early_stage/MAXENT/formosanus_jacknife_test.png]

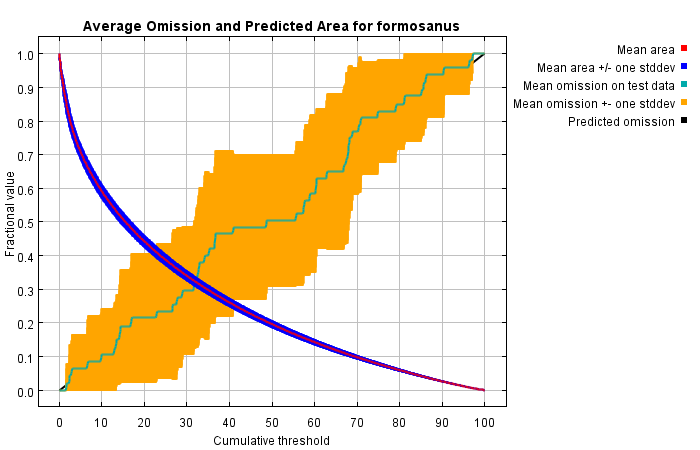

Supplement: Supplemental Information 6 [file peerj-11-14644-s006.zip › SDM/early_stage/MAXENT/formosanus_omission_past_2012.png]

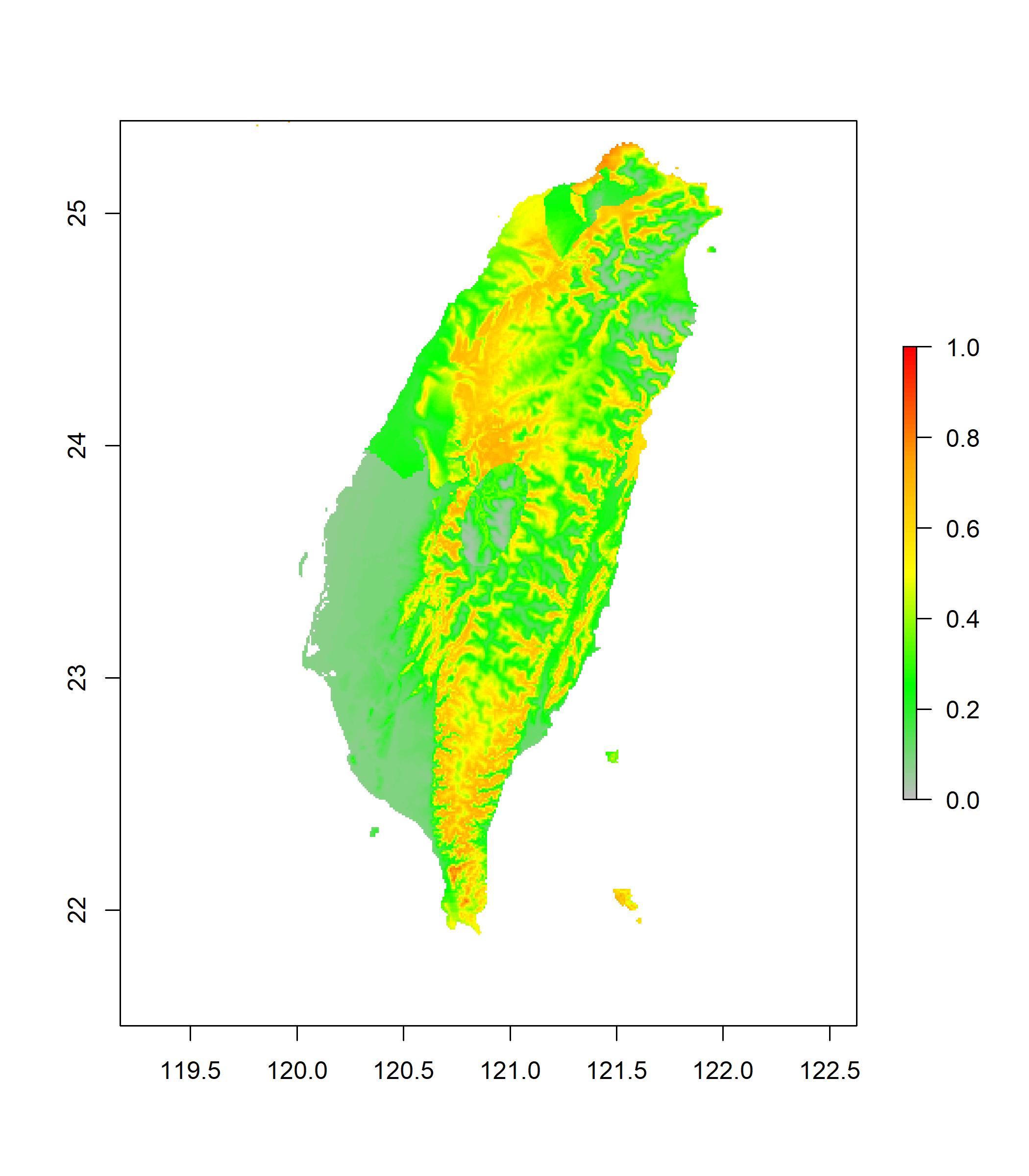

Supplement: Supplemental Information 6 [file peerj-11-14644-s006.zip › SDM/early_stage/MAXENT/formosanus_past_2012.jpg]

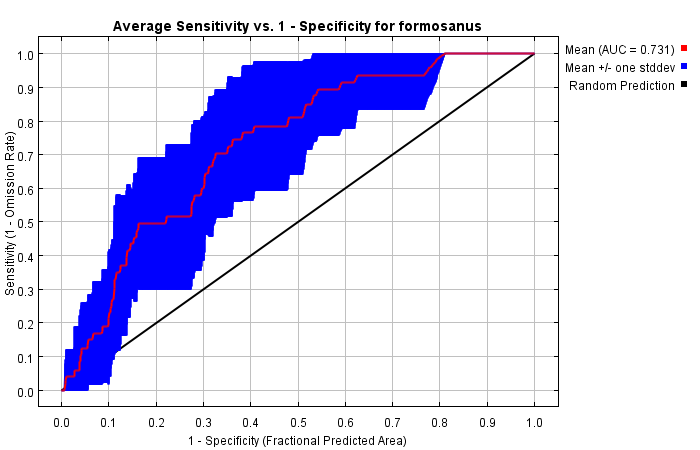

Supplement: Supplemental Information 6 [file peerj-11-14644-s006.zip › SDM/early_stage/MAXENT/formosanus_roc_past_2012.png]

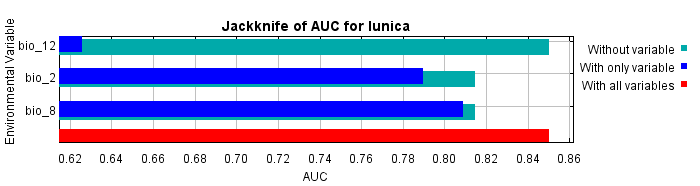

Supplement: Supplemental Information 6 [file peerj-11-14644-s006.zip › SDM/early_stage/MAXENT/lunica_jacknife_auc.png]

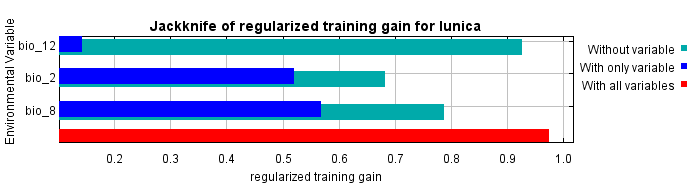

Supplement: Supplemental Information 6 [file peerj-11-14644-s006.zip › SDM/early_stage/MAXENT/lunica_jacknife_past_2012.png]

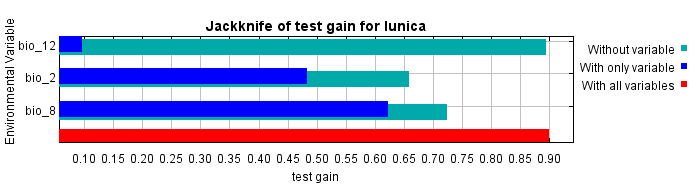

Supplement: Supplemental Information 6 [file peerj-11-14644-s006.zip › SDM/early_stage/MAXENT/lunica_jacknife_test.png]

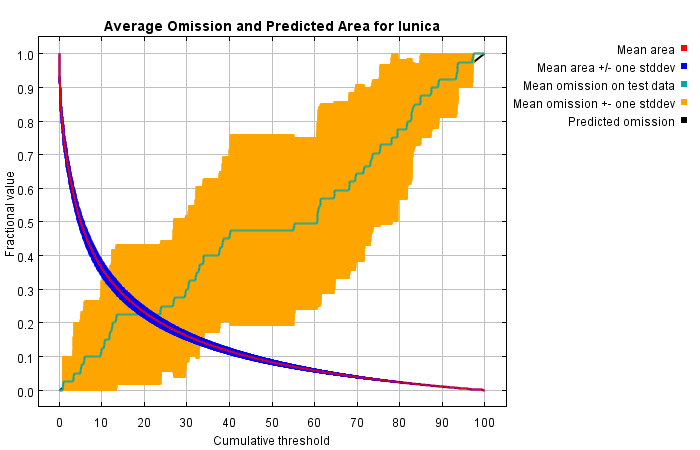

Supplement: Supplemental Information 6 [file peerj-11-14644-s006.zip › SDM/early_stage/MAXENT/lunica_omission_past_2012.png]

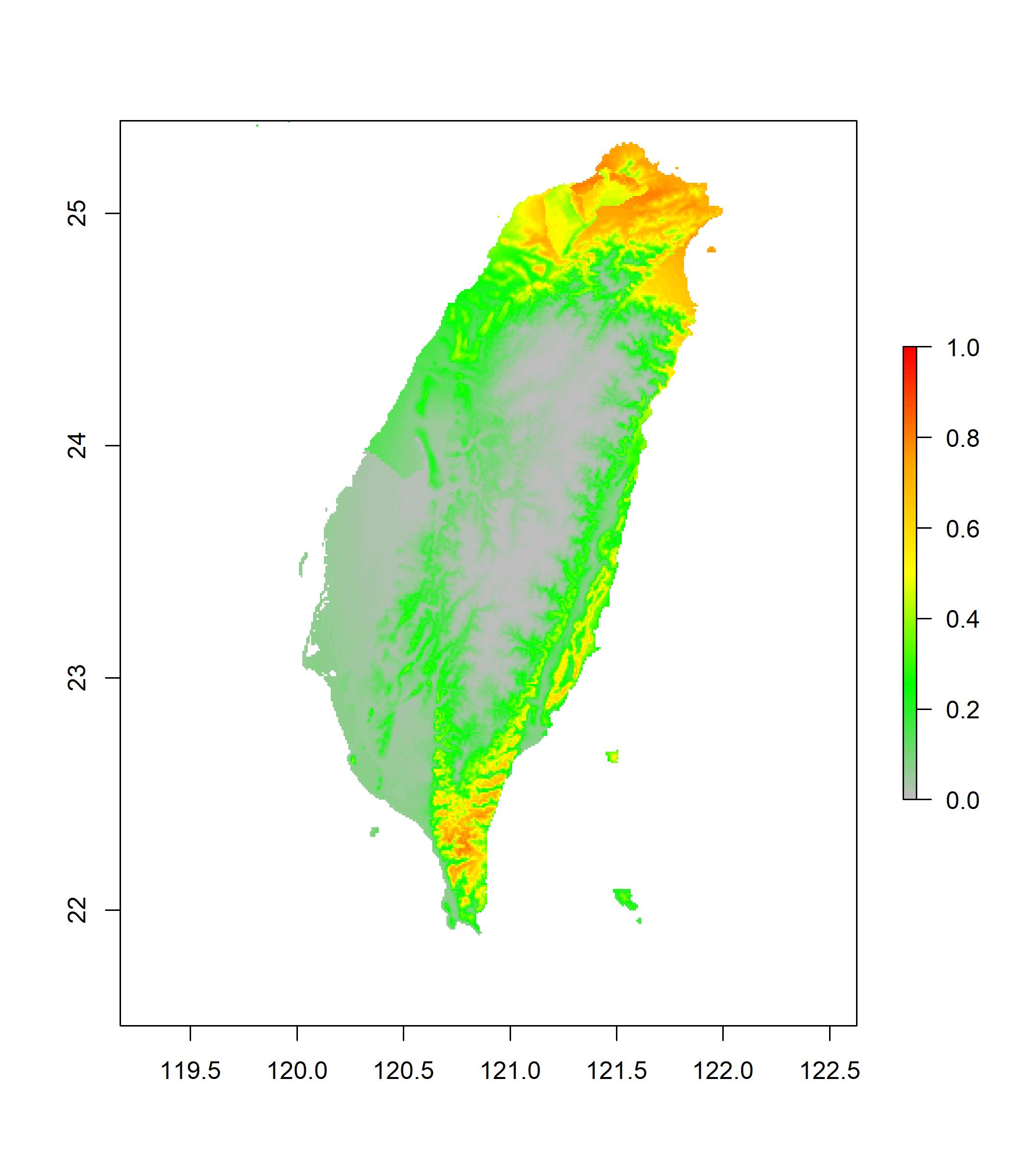

Supplement: Supplemental Information 6 [file peerj-11-14644-s006.zip › SDM/early_stage/MAXENT/lunica_past_2012.jpg]

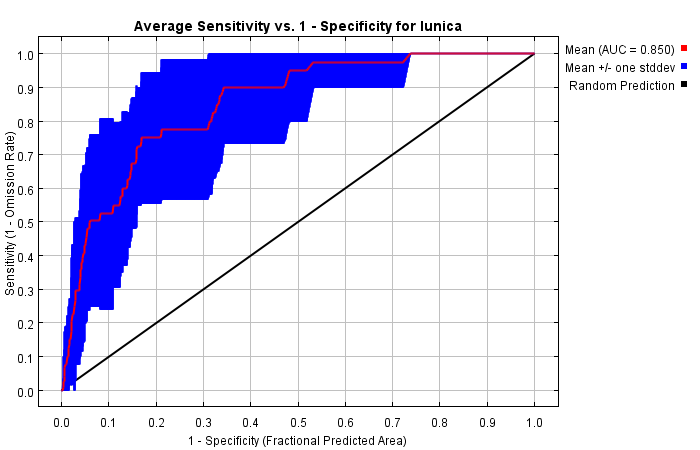

Supplement: Supplemental Information 6 [file peerj-11-14644-s006.zip › SDM/early_stage/MAXENT/lunica_roc_past_2012.png]

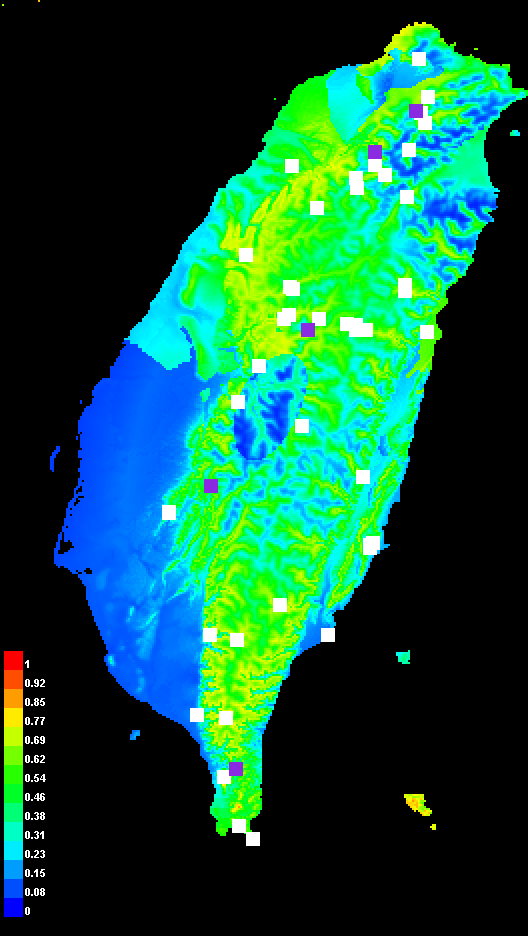

Supplement: Supplemental Information 6 [file peerj-11-14644-s006.zip › SDM/early_stage/MAXENT/past_2012_output/plots/formosanus_0.png]

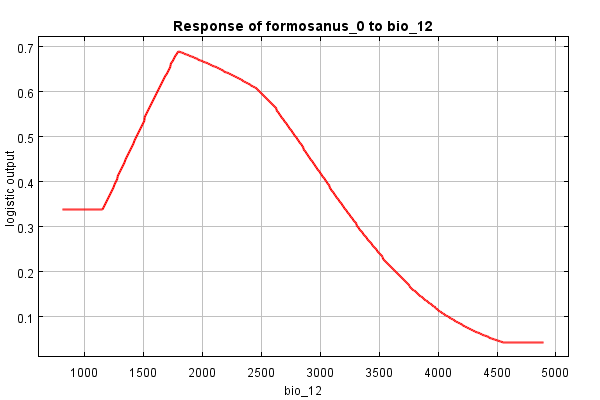

Supplement: Supplemental Information 6 [file peerj-11-14644-s006.zip › SDM/early_stage/MAXENT/past_2012_output/plots/formosanus_0_bio_12.png]

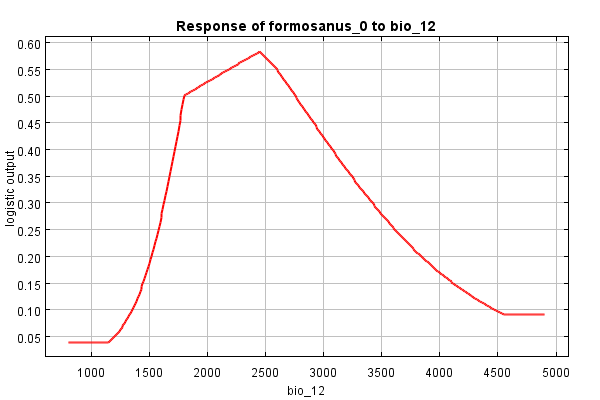

Supplement: Supplemental Information 6 [file peerj-11-14644-s006.zip › SDM/early_stage/MAXENT/past_2012_output/plots/formosanus_0_bio_12_only.png]

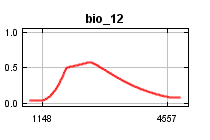

Supplement: Supplemental Information 6 [file peerj-11-14644-s006.zip › SDM/early_stage/MAXENT/past_2012_output/plots/formosanus_0_bio_12_only_thumb.png]

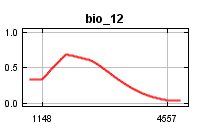

Supplement: Supplemental Information 6 [file peerj-11-14644-s006.zip › SDM/early_stage/MAXENT/past_2012_output/plots/formosanus_0_bio_12_thumb.png]

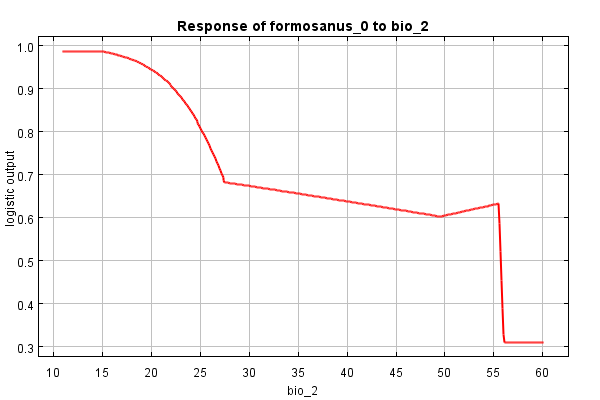

Supplement: Supplemental Information 6 [file peerj-11-14644-s006.zip › SDM/early_stage/MAXENT/past_2012_output/plots/formosanus_0_bio_2.png]

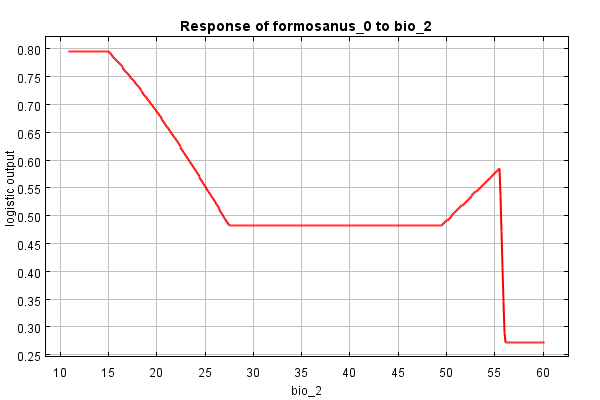

Supplement: Supplemental Information 6 [file peerj-11-14644-s006.zip › SDM/early_stage/MAXENT/past_2012_output/plots/formosanus_0_bio_2_only.png]

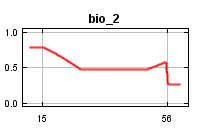

Supplement: Supplemental Information 6 [file peerj-11-14644-s006.zip › SDM/early_stage/MAXENT/past_2012_output/plots/formosanus_0_bio_2_only_thumb.png]

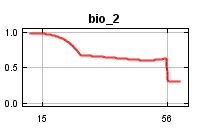

Supplement: Supplemental Information 6 [file peerj-11-14644-s006.zip › SDM/early_stage/MAXENT/past_2012_output/plots/formosanus_0_bio_2_thumb.png]

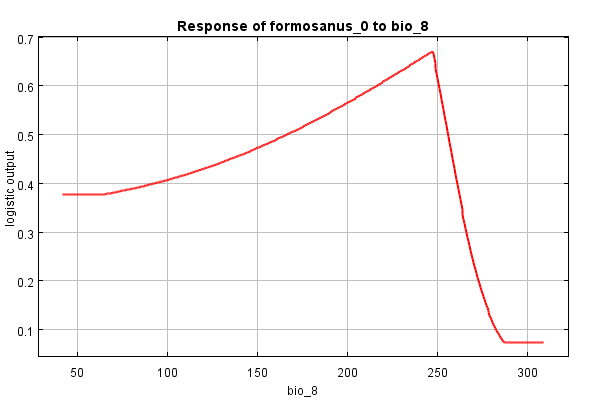

Supplement: Supplemental Information 6 [file peerj-11-14644-s006.zip › SDM/early_stage/MAXENT/past_2012_output/plots/formosanus_0_bio_8.png]

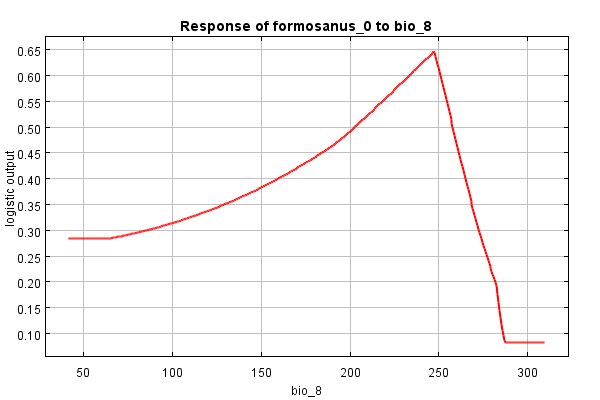

Supplement: Supplemental Information 6 [file peerj-11-14644-s006.zip › SDM/early_stage/MAXENT/past_2012_output/plots/formosanus_0_bio_8_only.png]

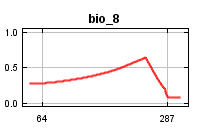

Supplement: Supplemental Information 6 [file peerj-11-14644-s006.zip › SDM/early_stage/MAXENT/past_2012_output/plots/formosanus_0_bio_8_only_thumb.png]

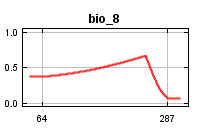

Supplement: Supplemental Information 6 [file peerj-11-14644-s006.zip › SDM/early_stage/MAXENT/past_2012_output/plots/formosanus_0_bio_8_thumb.png]

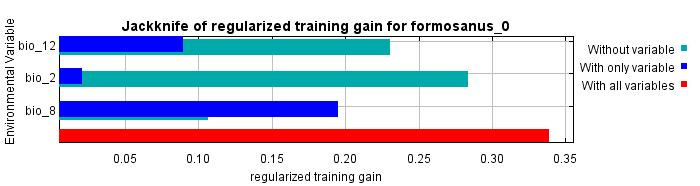

Supplement: Supplemental Information 6 [file peerj-11-14644-s006.zip › SDM/early_stage/MAXENT/past_2012_output/plots/formosanus_0_jacknife.png]
